# Psychosis Without Meaning: Creating Modern Clinical Psychiatry, 1950 to 1980

**DOI:** 10.1007/s11013-021-09744-3

**Published:** 2021-08-18

**Authors:** Joel T. Braslow

**Affiliations:** 1 Department of Psychiatry and Biobehavioral Sciences, UCLA, B7-435 Semel, Box #62, 760 Westwood Plaza, BOX 951759, Los Angeles, CA 90095 USA; 2 Department of History, UCLA, Los Angeles, CA USA; 3 Center for Social Medicine and Humanities, Jane and Terry Semel Institute for Neuroscience and Human Behavior, David Geffen School of Medicine, UCLA, Los Angeles, CA USA

## Abstract

Over the last fifty years, American psychiatrists have embraced psychotropic drugs as their primary treatment intervention. This has especially been the case in their treatment of patients suffering from psychotic disorders such as schizophrenia. This focus has led to an increasing disregard for patients’ subjective lived-experiences, life histories, and social contexts. This transformation of American psychiatry occurred abruptly beginning in the late 1960s and 1970s. My essay looks the ways these major transformations played themselves out in everyday clinical practices of state hospital psychiatrists from 1950 to 1980. Using clinical case records from California state hospitals, I chronicle the ways institutional and ideological forces shaped the clinical care of patients with psychotic disorders. I show there was an abrupt rupture in the late 1960s, where psychiatrists’ concerns about the subjective and social were replaced by a clinical vision focused on a narrow set of drug-responsive signs and symptoms. Major political, economic, and ideological shifts occurred in American life and social policy that provided the context for this increasingly pharmacocentric clinical psychiatry, a clinical perspective that has largely blinded psychiatrists to their patients’ social and psychological suffering.

## Introduction

Since the late 1960s and 1970s, American psychiatry has undergone dramatic changes. Though psychoanalytic and psychodynamic thought and practice dominated American psychiatry throughout the 1950s, it was a short-lived hegemony, replaced by growing faith in biological explanations of disease and an increasing reliance on psychotropic drugs. The National Institute of Mental Health (NIMH) research investment portfolio underwent a similarly drastic transformation in which research funding the social, psychological, and cultural dimensions of mental illness was dramatically curtailed from the late 1970s and early 1980s onward, replaced by increased funding into the biological aspects of mental illness. Accompanying this shift and, aided by it, the field of neuroscience grew at an extraordinarily rapid pace, producing spectacular discoveries in the fundamental understanding of the molecular biology, circuitry, and genomic regulation of the brain. But perhaps the most dramatic change since the 1960s has been complete dismantling of the U.S. system of care for those with serious mental illness. Peaking in 1955 with a resident patient population of over 550,000, state psychiatric hospitals had provided the vast majority of psychiatric care for over a century and, though already declining in size, disgorged most of their charges between 1970 and 1980.

At first blush, these dramatic changes look like progress fueled by science and effective interventions. “For the first time in its long and notorious history,” according to a former President of the American Psychiatric Association and current chair of a major academic psychiatry department, “psychiatry can offer scientific, humane, and effective treatments to those suffering from mental illness (Lieberman and Ogas, 2015, p. 10).” With a history of lobotomy and a host of other “heroic” and terrifyingly dangerous and infamous treatments in the profession’s rearview mirror, it is understandable that many view the present as progress against a vilified past. “The past two hundred years,” the former APA President continued, “the history of psychiatry has been characterized by long stretches of stagnation punctuated by abrupt and transformative changes—many of which, regrettably, were not for the better. But we have entered a period of scientific advances that will produce a stream of innovations more dazzling than any that have come before (p. 306).” This optimism is understandable as the millennium ushered in a new era of blockbuster psychiatric drugs that proved fabulously profitable for the pharmaceutical industry and that, in the case of Prozac, made sufferers of psychiatric ills “better than well” (Kramer 1993).

Yet, if one pokes just slightly beneath this rosy surface, one finds a much more complicated and darker picture. Most tragically for those with serious mental illness and most glaringly obvious has been the failure of our public mental health care system in the wake of deinstitutionalization. Irrespective of how effectively psychotropic drugs alleviate symptoms, they clearly do not provide food, clothing, shelter, significantly improved functioning, and a meaningful life. Far more individuals with serious mental illness now cycle through homelessness and incarceration in U.S. jails and prisons than occupied state hospitals at their mid 1950s peak. And, since the 1960s, the life expectancy gap between those with serious mental illness and rest of the population progressively widened. Today, an individual diagnosed with schizophrenia is likely to die 15–20 years prematurely.

At the same time, while the last fifty years have witnessed an unprecedented explosion in facts about the brain, this has not translated into enduring biological explanations of psychiatric disease. If anything, the biological sciences have undermined simple causal explanations of psychiatric disease. Complicating matters even more, new powerful genetic technologies have shaken the biological (or, at least, genetic) distinctiveness of the major psychiatric diagnostic categories. By the second decade of the twenty-first century, the sheen of the new antipsychotic and antidepressant drugs had worn off, revealing drugs that worked no better (but cost vastly more) than those discovered in the 1950s and 1960s.

Psychiatry is failing our sickest and most vulnerable patients. As a psychiatrist who treats primarily patients with psychotic disorders, I experience nearly daily my impotence to treat the core symptoms of my patients’ illnesses. Just as auditory hallucinations, delusions, and moments of motoric fury are symptoms of psychiatric disease, so too are the more visible social markers such as abject poverty, social isolation, homelessness, and a whole host of signs and symptoms that lead to arrest and incarceration. Nevertheless, I am largely trapped in a system of care that provides little leeway in providing the kind of solace, care, and treatment that, I believe, most psychiatrists would agree is genuinely adequate. Out of necessity, since nearly all I have to offer my patients is a prescription for a psychotropic drug (most commonly an antipsychotic), my relationship with my patients orbits nearly exclusively around the suffering that my drugs might help alleviate and the suffering that my drugs almost always inflict. In the case of the new generation of antipsychotic drugs, I am constantly worried that I may be contributing to my patients shortened life span as they balloon in weight and lipids clog their arteries.

As with most psychiatrists, I intuitively grasp that psychiatric disease is simultaneously biological, psychological and social. Yet, it is nearly impossible for me not to shove to the periphery of my clinical vision concerns about my patients’ social and experiential realities. If we take seriously that contemporary clinical psychiatry is the product of an increasingly better understanding of psychiatric disease and increasingly more effective interventions, we take comfort in the belief that we are on the right road, and just around the corner we will find ourselves in a “period of scientific advances that will produce a stream of innovations more dazzling than any that have come before.”

Others, including myself, are less optimistic. Andrew Scull, looking back over the last fifty years of American psychiatry, sees a profession undergoing a deepening crisis, largely of its own making (Scull 2021). He writes, “where once the American professional elite embraced Freudian ideas, for nearly a half-century it has bet on biology, and the wager has mostly turned up snake’s eyes.” While psychiatry has much to account for, I wonder whether clinical psychiatry’s narrowed vision can be chalked up to professional self-interest. Just as Freud made the Western world question the stories we tell ourselves about our motivations, I am skeptical that psychiatrists and their pharmacocentric vision of the world is entirely of their own making or, for that matter, the making of the pharmaceutical industry.

To better understand why psychiatry is in such an odd predicament, where we now often ignore the most obvious forms of our patients’ suffering and convince ourselves that our clinical practices and responsibilities can legitimately remain circumscribed by a narrow set of symptoms and imperfect medications, I want to take us back to the scene of the crime, a moment when clinical psychiatric care took an abrupt turn. Our story will begin with state hospital physicians and their patients in the 1950s. We will then follow our protagonists as their world begins to unravel and they find themselves in a decidedly harsher reality in the late 1960s and 1970s. Though I believe more expansive ways of caring for and treating individuals with serious mental illness are more humane and ethical than an approach that reduces psychological suffering to a biological dysfunction that requires a biological solution, I am not intending this history as necessarily a defense of state hospital care, even if it appears more humane than we might have imagined when we look more closely at actual clinical practices during this period of time. Instead, my aim is to understand how everyday contexts, resources, and constraints shaped the ways in which psychiatrists understood their patients’ ills, listened to them, and treated them. In other words, by looking at what actually went on between psychiatrists and patients we might better understand the forces that have created our contemporary way of caring for those with serious mental illness.

I base this examination on patient case files from California state hospitals of patients treated between the 1950s and 1980s.^1^ Written by psychiatrists, these case files give us first-hand accounts of what psychiatrists found noteworthy and, as we follow their chronicles, we will see more directly the ways in which clinicians made sense of and rationalized their clinical treatment of their patients.

## Part I: Psychosis, Meaning, and the Social World (1950–1970)

Over the last century, a variety of social critics, journalists, former patients, and physicians have written accounts of state hospitals in order to reveal patient abuse, systematic dehumanization and neglect, massive overcrowding, and unspeakably horrific living conditions (e.g., Beers 1908; Carlisle 2010; Ward 1946; Deutsch 1948; Goffman 1961; Maisel 1946). Nevertheless, state hospital conditions improved dramatically after World War II. In California, for example, the budget of the Department of Mental Hygiene increased by about four-fold in the decade following the War. This allowed the state to repair crumbling buildings and build new ones. By 1955, with the post-War construction boom, California state hospital administrators had nearly entirely vanquished overcrowding, reducing it to 1.8 percent (California 1955). At the same time, the number of attendants and physicians relative to patients nearly doubled (Braslow 1997). Improved conditions resulted in shorter hospitalizations, even for patients diagnosed with schizophrenia. For the 988 patients admitted in 1949 with a diagnosis of schizophrenia, 63% were discharged within 12 months. In 1956, 79% of the 1,577 patients admitted with a diagnosis of schizophrenia were discharged within 12 months (Epstein and Morgan 1961).

Of course, none of this is to say state hospital care was entirely benign. Throughout the 1950s (and, in fact, over the course of their entire history), periodic scandals of patient abuse leapt across the state hospital grounds and onto the front pages of California newspapers. In one of the most widely publicized reports of patient abuse, five different state investigative teams were dispatched to Modesto State Hospital to investigate charges of “brutality and inhumane treatment” (Los Angeles Times, 1956a, b). Yet episodic reports of horrific abuse ranging from murder to physical brutality and humiliation did not undermine the legitimacy of state hospital care. For example, Edmund “Pat” Brown during his tenure as California Attorney General between 1950 and 1958 was among the most vocal critics of conditions in California State Hospitals declaring them “a scandal to a state possessing such wealth” (*The Los Angeles Times*, December 15, 1956). He went on to claim that “instances of brutality and mistreatment in our state hospitals are not uncommon occurrences.” Nevertheless, Brown defended the principle of state hospital care even as he vigorously prosecuted individuals responsible for abusive conditions. “It isn’t the system that’s wrong,” according to Brown, “it’s the administration of the system” (*The Los Angeles Times*, September 11, 1956). It would take at least another decade before politicians would seriously begin to question the wisdom of state hospitals.

Of course, physical violence and overt abuses of power by attendants and psychiatrists were not the only potential horrors of 1950s state hospitalization. As Erving Goffman famously argued, psychiatric hospitalization created its own nightmares of humiliation, mortification, loss, and various forms of psychological and physical subjugation in the course of a patient’s “moral career” as a psychiatric patient (Goffman 1961). Goffman’s account (along with numerous other sociological and ethnographic accounts) of “total institutions,” ranging from concentration camps, to prisons, to mental hospitals has had a profound effect on the ways in which state psychiatric institutions were and are imagined. Along with a diverse range of antipsychiatric literature of the 1960s and 1970s, these critiques of life within psychiatric institutions helped to justify the dismantling of one of the most visible signs of the welfare state. Goffman’s ethnographic observations of “inmate” life in Washington D.C.’s St. Elizabeths Government Hospital for the Insane depict psychiatric hospitals as powerful machines for the dehumanization of individuals. Conflating prisons, concentration camps, and psychiatric hospitals, Goffman’s “total institutions” are the antithesis of his idealized world of life under 1950s American capitalism. “In our society,” he writes, “they [total institutions] are the forcing houses for changing persons; each is a natural experiment on what can be done to the self” (p. 12). From admission to eventual discharge, the psychiatric “inmate” undergoes regimentation, tyrannical disciplinary control in the name of treatment (e.g. electroshock as a means of behavior control), and a series of humiliations all aimed at creating compliance and a new kind of self.

Goffman’s psychiatric hospitals have little to recommend as a shelter for an individual whose psychosis has made life impossible outside of the institution. “However harsh the conditions of life in total institutions, harshness alone cannot account for this sense of life wasted.” It is the illusion of treatment that adds to the depredations inflicted upon the individual by the hospital:

One of the virtues of the doctrine that insane asylums are treatment hospitals for sick people is that inmates who have given up three or four years of their life to this kind of exile can try to convince themselves they have been busily working on their cure and that, once cured, the time spent getting cured will have been a reasonable and profitable investment.

This sense of dead and heavy-hanging time probably explains the premium placed on what might be called removal activities, namely, voluntary unserious pursuits which are sufficiently engrossing and exciting to lift the participant out of himself, making him oblivious for the time being to his actual situation. If the ordinary activities in total institutions can be said to torture time, these activities mercifully kill it. (pp. 68–69)

As we will see below, the everyday world of state hospitals could and often did look very different from these depictions of state hospitals as factories of psychological and physical torture.

Looking back since the publication of Goffman’s *Asylums*, we see that there are far worse alternatives to state hospitals. And, with hindsight, we can see that critiques of state institutions, regardless of their truth, ironically and tragically did not aid in the liberation of those with serious mental illness but, instead, by undermining the legitimacy of state hospitals, helped provide the rhetorical justification for a far more brutal system. If there could be a dark side to the “total institution,” there is another side as well, one that requires a more nuanced vision of psychiatric care and underlines the importance that everyday institutional realities play in the “mortification” of patients as well in providing solace and asylum.

### Social and Psychological Meaning in Everyday Clinical Practice

Just as we should take seriously (and critically) the potential and actual dangers of state hospitals, even in that hopeful moment of the 1950s, we should take seriously (also skeptically) what hospital administrators had to say about their institutions. Entering the 1950s, hospital administrators turned the totalizing nature of care into an exuberant and expansive therapeutic mission. The Director of the California Department of Mental Hygiene wrote in 1952, “Everything done to, for, or around a mental patient in a California hospital can be considered as therapy since therapy is the ultimate purpose of everything done” (California 1952). There is truth to this claim if one were to judge from annual reports. The 1950 report lists the following well-known and now largely infamous therapies: electroshock therapy, lobotomy, sterilization, and hydrotherapy. In greater detail, the report also lists additional therapies that psychiatrists employed with nearly all patients rather than the relatively few patients who received therapies such as lobotomy and sterilization. These additional treatments included industrial therapy, occupational therapy, recreational therapy, bibliotherapy, music therapy, and beauty shop therapy. While many of these “therapies” provided critical patient labor for maintaining these massive institutions (Camarillo State hospital, for example, had a peak resident patient population of over 7000 patients), psychiatrists took them seriously as treatment interventions, even if they could be read in more sinister ways by others or by patients themselves. Industrial therapy, the report tells us, “is a vital part of the treatment program for it is a recognized fact that idleness is detrimental and that a reasonable amount of productive labor is beneficial to mental well-being” (California 1950). In the case of industrial therapy, about half of all male patients and about a third of female patients engaged in this form of treatment.

Dividing “therapeutic activities” into “nonmedical” and “psychiatric and related medical therapies,” the annual reports of the early 1950s classified psychotherapy under the heading of “psychiatric and related medical therapies.” Not only is psychotherapy listed first among the medical interventions, the annual reports devoted more space to its discussion than any other form of treatment, medical or otherwise. “There is,” the Medical Superintendent wrote, “an acknowledged need for this type of therapy, on both a group and individual basis in the effective treatment of mental illness” (California 1950). He continued:

Fundamentally, the purpose of psychotherapy is to assist the patient in gaining insight that will enable him to discover for himself the exact nature of his difficulties, the factors leading to his present attitudes, and the reasons why they should be changed.

Surprisingly, given the size of the patient population, the clinical case files suggest that physicians genuinely tried to engage their patient’s psychotherapeutically, even those who were psychotic. Let us start with a randomly chosen case file of a patient admitted in 1950. Claude James was one of over 1100 individuals admitted to Stockton State Hospital that year. According to the admission papers, the police observed Claude running nude through the streets of Sacramento and screaming “I am god in the sun. The devil is in everything. Look out for snakes.” His hospitalization began traumatically. Not only did Claude require shackles for the hour trip from the Sacramento jail to Stockton, but once he arrived at the Receiving Building, Claude continued to rage. The admitting note reads:

Patient was about to be interviewed by Dr. Adams and started yelling and when attendants attempted to place him in the side room, started kicking and fighting attendants and other patients. When they attempted to place him in side room attendant Summers was kicked and bit and Attendant Ponder was scratched and bit.

Once the attendants succeeded in strapping each limb in leather restraints, tied securely to the four corners of the gurney, the ward physician entered the room noting,

His hair was mussed and he had a couple days growth of beard. The patient was lying very rigid, breathing deeply and shouting when the interviewer entered the room. The patient was commanding and hostile…He would holler and shout and then talk in a strained manner. After about a half hour of commanding, the patient changed his attitude slightly and became confiding. His face was tense, he would open his eyes wide, dilate his nostrils and grimace. The patient would sit up and strain his muscles until he would tremble.

A stenographer provided a four-page transcript of the remaining interview. For the next hour, Claude spoke in long, often convoluted sentences, about God, the devil, death, sin, and his mother who worked in the “shit house” and was in league with the devil. “The devil had trickest me many times…(looks up at the ceiling he shouts) LEAVE THE ROOM YOU DEVIL I SEE YOU ON THE CEILING! (Dr. Gorman asked ‘what is this place?’) This is the workshop of the devil, this is the death house.”

Claude’s agitation finally reached a crescendo requiring chemical intervention. The stenographer notes the appearance of an attendant with a hypodermic filled with sodium amytal. With the assent of Dr. Gorman, he then injected the barbiturate into Claude’s arm. Claude’s only response was, “You can put it in, it will not stop the tongue.” The barbiturate apparently worked as the stenographer notes that the patient had calmed down and appeared drowsy. The remainder of the interview went as follows:

**Claude**: Do you think I’m Crazy?

**Doctor**: No, I do not think you are crazy.

(The patient got a warm smile on his face)

**Claude**: Shake the hand, feel the warmth.

(The patient then went into a drowsy comma).

With Claude no longer responsive and the interview effectively ended, the attendant wheeled Claude back to his ward.

Over the next few weeks, Claude underwent a course of electroshock treatment, hydrotherapy, and psychotherapy. To assess his progress and ascertain a precise diagnosis, his attending psychiatrist convened a “Clinical Conference,” a ritual in which all newly admitted patients were interviewed by several staff psychiatrists (in Claude’s case there were 5 present) with a stenographer transcribing the patient interview and subsequent discussion between the assembled physicians. Claude had calmed down significantly since his first few days in the hospital and no longer ranted about God, the devil, and his possessed mother. At the same time, he had little insight into the events that brought him into the hospital. When asked by Dr. Gorman whether there was “anything wrong with your mind?” he replied that “I may have had a little fever at the time. My mind is as good as it ever was.” Unsatisfied, Dr. Weiss persisted: “All this business about God and snakes, does it represent any illness?” Again, Claude answered, unwilling to see himself as psychotic: “I was reading the Bible.”

After dismissing Claude, the physicians had the task of arriving at a diagnosis.

**Dr. Weiss**: The mother says he is a quiet boy, never fights or quarrels.

**Dr. Brunnell**: Schizophrenia, catatonic type.

**Dr. Batko**: Schizophrenia, mixed type.

**Dr. McCullough**: Mixed. He will turn out to be paranoid.

**Dr. Doody**: Catatonic.

**Dr. Galioni**: Catatonic.

**Dr. Weiss**: Dementia Praecox, catatonic type. He should have EST.

At first glance, there is very little to distinguish Claude’s psychiatrist from how a psychiatrist today might have approached Claude’s disease. Then, as now, little has changed about the kinds of behaviors considered to be psychotic and central to a diagnosis of schizophrenia or dementia praecox (used interchangeably with schizophrenia in the early 1950s). While psychiatrists today might prescribe electroshock therapy after trying numerous antipsychotic drugs, Claude’s psychiatrists would have to wait another five years for the first effective antipsychotic drug (chlorpromazine, brand name Thorazine) to be available. But, on the surface, one could imagine that the logic of today is quite similar as it was in the 1950s where a set of psychotic symptoms (such as delusions, auditory hallucinations) signify an underlying biological disorder requiring a biological solution.

If we probe deeper, however, we see that Claude’s psychiatrists had a significantly more complex notion of Claude’s illness. Claude’s psychotic symptoms and signs signified more than an underlying biological disease. His psychiatrists believed that his fantastical and terrifying delusions about the devil and his mother had psychological and historical meaning that not only helped to explain the cause of his psychosis, but had therapeutic value. They extensively probed his early childhood. Impoverished and often desperate, his parents hit him frequently and, when he managed to avoid their frequent beatings, he felt neglected and alone. His physicians paid particular attention to his psychosexual development. “The patient said, when he was very young, he saw his parents have intercourse many times and many times he would lie awake all night listening to his parents argue because of sex.” His physicians gave these early experiences a causal role in Claude’s psychosis. They described his early experiences as psychological pathogens, lurking in his past and slowly eating away at him until they erupted into psychosis.

The psychiatrists carefully chronicled what they considered most important and pathogenic. His “early home life [of] destitution,” forced to work at the age of 14, “hired out for 50 cents a day and worked from daylight to dark driving mules around plowing fields,” and, most importantly, the “many traumatic incidences involving sex and parental relationships” gave psychological meaning to his disease. “She [his mother] never done nothing for me but brought me into the world,” Claude told his ward physician. He continued, “She let me run around with shit in my pants. Box my ears until I was deaf and I would lose my senses.” In what his physician thought was most meaningful, Claude said, “I was seeking love, she did not have any for me.” Suggesting that something recently had triggered his psychosis, his physician asked whether he frequented sex workers. “The last time I went to one was three month ago,” he confessed and then began to cry. “I loved them…I asked many of them to marry me, to love them, they said maybe.” Then, echoing his earlier comments about his mother and what he had experienced as her having “done nothing for me,” his love turned dark: “They would suck the dick with their mouth, eat the shit from the ass. They would do anything for money.” For Claude’s physicians, electroshock therapy could do nothing for what genuinely gnawed at Claude and ultimately proved the most pathogenic. While electroshock therapy may have been useful for his acute symptoms, his psychiatrists believed that psychotherapy would get at the core of his psychosis by helping him forge connections between his pathogenic past and his psychotic signs and symptoms.

Claude’s hospitalization was short, lasting only three and half months. He improved rapidly, receiving psychotherapy, electroshock therapy, occupational therapy, and attending the various entertainments, such as movies, picnics, and field trips into the surrounding city of Stockton. Brought before the “Clinical Conference” a few days before his discharge, the physicians made certain that he was no longer psychotic and that his mother was willing to take him back. Curious about what accounted for his improvement, one of the physicians present asked “which helped the most, EST or group therapy?” Claude replied, “Well, I don’t know—they both helped me.” Of course, we cannot be certain whether Claude simply wanted to please his physicians and gave them an answer he knew they wanted to hear. Nevertheless, we do know that his physicians tried to give meaning to his psychosis, a meaning embedded in his past and present desires that, at least from the physicians’ perspectives, required both somatic and psychological interventions.

### Antipsychotic Drugs and Psychosis

The same year Claude was admitted to Stockton, 1950, the French pharmaceutical company Rhône-Poulenc, synthesized RP 4560 (chemical name chlorpromazine). Chlorpromazine, which was marketed as Thorazine in the United States, inaugurated an unprecedented and massive psychopharmacological revolution that continues to reverberate through present-day psychiatry. Indeed, many have assumed that our contemporary dependence on psychotropic drugs inevitably followed from their effectiveness (even if partial, at best). As a corollary, psychiatrists often see the fact that a drug’s capacity to alter a particular behavior or experience (such as an auditory hallucination) as proof that the psychiatric disease is fundamentally biological in nature and that the social and psychological are largely epiphenomenal, playing a secondary role. A close look at the ways in which psychiatrists used antipsychotic drugs reveals that there was nothing inevitable about the specific ways in which physicians would eventually use these drugs beginning in the 1970s. As we will see, the social, institutional, political, and ideological contexts of care mattered as much, if not more so, than the actual biological effects of the drugs in shaping psychiatrists’ growing reliance on them as their most important interventions.

A synthetic phenothiazine, RP 4560 was a product of the company’s efforts to create centrally acting antihistamines. Having given the drug to a number Parisian clinicians to try on their patients, some observed that the drug calmed psychotically agitated patients (Swazey 1974). By 1952, clinicians found the drug useful for a number of conditions and Rhone Polenc marketed it as Largactil, to reflect its many potential applications. In 1952, Smith Kline French purchased the North American rights to the drug and, in 1954, after receiving FDA approval, the company marketed it under the brand name of Thorazine.

Early on, Smith Kline French recognized the size of the potential psychiatric market. Over a half a million captive potential consumers housed in the United States sprawling state hospital system, no doubt, motivated Smith Kline French initially to provide Thorazine free of charge to state hospitals so physicians could judge for themselves the effectiveness of this new pharmaceutical agent. Ultimately, affirming the published literature, practitioners found chlorpromazine enormously useful in quelling many psychotic symptoms, especially agitation, combative and violent behavior, extreme anxiety, as well as overtly psychotic symptoms such as auditory hallucinations, disordered and chaotic thinking, and delusions. By 1955, Thorazine had become an early blockbuster drug with profits of $75 million (Overholser 1956: 198). Over the next decade, Thorazine sales drove the company’s rapid growth, from net sales of $53 million in 1953 to $347 million in 1970 (Scull 2015).

Though Heinz E. Lehmann first used the adjective “antipsychotic” in 1956, psychiatrists did not routinely use the term until the mid to late 1960s (Lehmann 1993: 300). When chlorpromazine entered the psychiatric market, clinicians (as well as the pharmaceutical companies) saw these drugs as all-purpose tranquilizers, albeit different than previous agents in that they caused less sedation. Reflecting the drugs’ clinical uses, psychiatrists used the terms major tranquilizers, ataractics, and neuroleptics. The term “major tranquilizer” was employed quite early on to distinguish these agents from the “minor tranquilizers” such as meprobamate (Miltown). It took nearly a decade of use and significant marketing efforts by drug companies to tip the balance in favor of these drugs acting specifically on psychosis. With these caveats in mind and to avoid confusion, I use the modifier “antipsychotic” to avoid using multiple terms for the same class of agents (Braslow and Marder 2019).

### Antipsychotic Drug Use in California State Hospitals

California state hospital psychiatrists first began experimenting with Thorazine in December 1954, seven months following FDA approval. By the end of 1955, all 13 of California state hospitals had begun using drug. Despite this, California state hospital superintendents remained cautiously optimistic, tempered with a healthy dose of skepticism. The Director of Mental Hygiene, Walter Rapaport, explained to a 1956 California Senate Committee that the glowingly positive reports in the popular media, as well as positive assessments in the scientific literature, should be looked at critically. The California Senate had convened the committee precisely because the popular press reports described Thorazine and the growing number of new tranquilizing agents as revolutionary new treatments for mental illness (Plumb 1955; Cant 1956; Pompian 1955). The fact that Rapaport had only requested $48,300 for fiscal year 1955–56 and $206,688 for the following fiscal year out of annual budgets of around $80,000,000 per year had led the legislators to worry that California state hospital physicians were failing to use the most modern and scientific treatments (California 1956: 25).

In the past, Rapaport and his colleagues had seen a number of “cures” enthusiastically embraced, promising wildly positive outcomes for desperate families and their tormented loved ones, only to prove to be the therapeutic equivalent of fools’ gold. Insulin coma therapy, introduced in the early 1930s, was among the most disappointing of the “great and desperate cures” introduced in the 1930s (Braslow 1997). In response to the Senators’ dismay that California state hospital physicians only sparingly used the new tranquilizers, Rapaport gave a short history lesson, “I must caution you Senators about 15 or 16 years ago, and I would recommend that if you have time you read some of the literature of 1938,1939, and 1940. They were curing this many patients with insulin in the schizophrenic group right here in the State of California” (California 1956: 49). He then added, “I would say that a lot of people who said that methanol was the final answer and insulin was the final answer, now would like to chop off a few of their words” (California 1956: 53).

In a series of public hearings in late 1955 and 1956, California state hospital superintendents summarized their experiences with the new tranquilizing agents, views that largely affirmed Rapaport’s cautious optimism that Thorazine was not curative, but a new useful addition to their therapeutic armamentarium. Summarizing the sentiments of many of his fellow psychiatrists, the former Superintendent of Stockton State Hospital, Rudolph Toller, said:

I feel the new drugs have a decided value in the treatment of psychiatric cases seen in the private practice of psychiatry. It is my opinion that while the drugs are not curative, they prepare the patient for other psychiatric techniques of treatment. The anxious and tense patient, for example, becomes more vulnerable to psychotherapy. The restless and agitated patient is quieted down and able to accept psychotherapy. (California 1956: 17)

It is worth stressing that the new psychotropic drugs were not initially framed as particularly revolutionary. Useful, yes. But, at least from this former administrator’s perspective, the drugs remained largely an adjunctive treatment to “other forms of psychiatric techniques.” Just as importantly, there was nothing in the nature of prescribing these new psychoactive drugs that necessarily dictated a particular way of understanding psychiatric illness. The fact that an agent acted through a biological mechanism did not in and of itself suggest that the illness was either solely biological in nature or that psychological and social interventions were less important.

In what follows, we will follow a couple of patients in their sojourns through California state hospitals so we can witness firsthand the ways psychiatrists in their everyday clinical care made sense of these new interventions. To foreshadow my argument, it was the structure of state hospital care that played a dominant role in structuring how antipsychotic drugs would be deployed and rationalized. What much of the antipsychiatric literature ignores or downplays is that these institutions were, at least in principle, designed, funded, and staffed with the aim of comprehensive care for those individuals whose family and friends had forsaken and who were far too impaired to survive employed and housed. These structural forces, as much as the drugs and their effects, dictated the meaning of psychosis and its treatment.

### Antipsychotic Drugs in Daily Practice, Part 1: Affirming the Social and Psychological

Albert Russo already had spent a relative brief stint at Stockton State Hospital, when in the fall of 1959, just after his twenty-first birthday, he again became psychotic. Still living at home, Albert was convinced he had been endowed with “magical powers” that would allow him to visit the moon. Not surprisingly, his parents expressed skepticism and a good deal of alam since his previous hospitalization had followed a similar episode. Along with his growing delusional fantasies, Albert had become “aggressive and belligerent.” Unable to keep him safely at home, they decided that they had no choice but to seek help. Cajoling him into their car, they drove him to the local county hospital. There, the examining physician transferred him to Stockton, diagnosing a psychiatric disorder that required longer and more intensive care than the county hospital could provide. Albert arrived at Stockton’s receiving hospital just as delusional and agitated as he had been at the county hospital. The psychiatrist had little trouble arriving at a diagnosis of schizophrenic reaction.

At the end of the nineteenth century, psychiatrists developed what would become a fairly standardized structure to their psychiatric histories, which has changed little, if at all, over the course of the twentieth century.  The typical psychiatric admission history contained the following headings: present illness, past illnesses, family history, personal history, mental status, formulation, and diagnosis. While the broad structure remained remarkably stable over time, psychiatrists’ narrative styles and the kinds of stories they told about their patients’ suffering did change over the course of the century. In the first few decades of the twentieth century, physician histories read more like natural histories charting the unfolding of an autonomous disease process that gradually revealed itself over the course of the patient’s illness. By the 1940s and 1950s, one can discern a marked change in the ways in which psychiatrists wrote about their patients.  Instead of only cataloguing the appearance of signs and symptoms, psychiatrists’ increasingly tried to fashion a causal story about their patients’ ills. In particular, psychiatrists increasingly wrote stories where their patients’ childhood experiences, losses, desires, failures, and psychological traumas played a necessary, if not sufficient, causal role in their diseases.  Albert’s psychiatrist leaves no doubt that he saw Albert’s disease as flowing directly out of his childhood relationships with his “passive” mother and “hostile” father:

In his family the father appears to be very rigidly and aggressively domineering and the mother appears to be a warm and loving, but ineffectual parent. There appears to be a great deal of conscious and unconscious hostility between these parents. Shortly after the patient’s older sister eloped, an action which the father firmly disapproved, and after the patient’s own failure to make an athletic team at school, his behavior became erratic, hyperactive, and less well organized in general. It seems possible that the patient is torn between the desire to act out his father’s hostility and the desire to be more passive or submissive like his mother.

Albert’s psychiatrist constructed a narrative of Albert’s disease that wove the parents’ internecine battles as the pathogenic nidus that eventually laid the groundwork for a “schizophrenic reaction.” The psychiatrist’s treatment recommendations reflected his analysis of Albert’s troubles; namely to make conscious his largely unconscious conflicts revolving around his relationships with his parents and the meanings these had for him. This would be possible through psychotherapy. Of course, we should be cautious as to what “psychotherapy” means in this context. The psychiatrist failed to jot down how often he met with Albert as well as what may have transpired in their meetings. Most likely, their sessions had little in common with psychodynamic, let alone, psychoanalytic psychotherapy. Instead, given the time constraints the psychiatrist faced in caring for a large number patients, he most likely met with Albert for short intervals, giving practical advice, playing a surrogate parent, and shoring up Albert’s sagging self-esteem.

The psychiatrist’s psychological explanation for Albert’s ills and his psychotherapeutic solution did not preclude him from prescribing antipsychotic drugs for Albert. Immediately upon Albert’s admission, the psychiatrist ordered Stelazine (trifluperazine), another antipsychotic drug that Smith Kline French began marketing in 1958 (Shorter 2005: 55). It is clear from the notes that the psychiatrist did not see the antipsychotic drug as central to Albert’s psychiatric treatment. Instead, the doctor used the medication as a practical and rapid solution to Albert’s anxiety. He did not describe it as a cure nor did the fact that the Stelazine was useful in diminishing Albert’s anxiety have implications for his understanding of Albert’s disease. What mattered to the psychiatrist was to sufficiently calm Albert so that he could engage in psychotherapy. “It is my impression,” the psychiatrist wrote, “that the patient is hungry for a relationship with a male in order to work through some of his difficulties he has with his father.” In preparing for his discharge, his psychiatrist worried that “the dominant father may object if he sees that the therapy is arousing the patient to rebel from his influence.” Stelazine offered a solution. He reasoned that if he continued to prescribe the medication for Albert, the father might not object to Albert’s psychotherapy: “the prescription of Stelazine, 5 mgs b.i.d., might serve as an incentive for cooperation of the family in having the patient continue coming to [therapy].”.

**Fig. 1:**
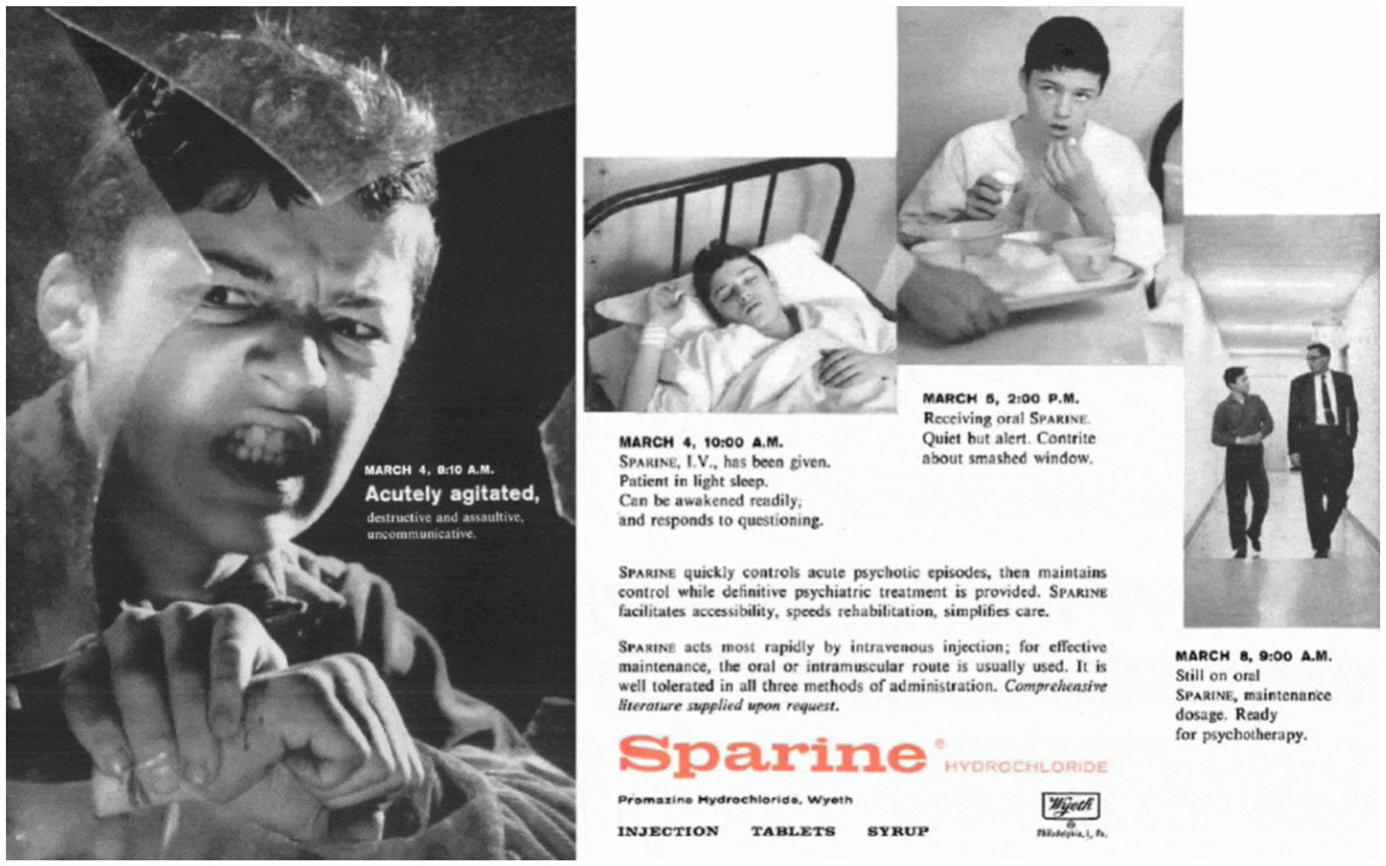
Sparine Advertisment, 1959

This use of psychotropic drugs was not unusual. A 1959 advertisement for the antipsychotic drug Sparine (promazine), Fig. 1, tells nearly an identical story to that of Albert’s (Sparine 1959). Like Albert, the faux patient is “acutely agitated, destructive and assaultive.” Sparine “quickly controls acute psychotic episodes,” the ad copy reads, “then maintains control while definitive psychiatric treatment is provided.” In an optimistic time course of 4 days, the ad shows a well-dressed, attentive young man looking deferentially at presumably his psychiatrist, “ready for psychotherapy.” The ad, as well as Albert’s case history, suggest that the early use of psychotropic drugs did not revolutionize in any simple way the understanding and treatment psychological distress, even when it came to psychotic patients. Instead, as we will see, what mattered most in shaping the meaning of disease and treatment was the context in which care took place.

### Consolation and Care

Throughout the nineteenth and first half of the twentieth century, state hospitals provided a safety net of last resort for those whose mental illness made it impossible to survive on their own.  All of the stuff that makes life possible outside of the hospital was the responsibility of the physicians within its walls.  As such, the claim that “Everything done to, for, or around a mental patient in a California hospital can be considered as therapy” underlines the historical contingency of treatment practices and the boundaries of psychiatric disease.  For state hospital physicians, responsible for all aspects of their patients’ lives,  disease its treatment easily traversed across the domains of biology, psychology, and social life. It is not surprising, then, that psychoactive drugs (especially given their limited efficacy) had little overall effect in altering the ways in which psychiatrists understood and treated their psychotic patients.

The totalizing nature of state hospitals, where physicians tried to regulate (even if for therapeutic ends) all aspects of patient life, did not dissuade patients from voluntarily admitting themselves into state hospitals.  Following the war, increasingly large numbers of individuals sought voluntary hospitalization. Embarking on a massive post-War construction spree, California nearly entirely vanquished overcrowding. At the same time, California state hospital administrators hired numerous psychiatrists, psychologists, and social workers who were awash in the growing cultural influence of psychoanalysis and psychotherapeutic approaches to mental ills. California state hospitals of the 1950s provided asylum not only for those involuntarily forced into the state hospital, but also a growing and significant number of individuals voluntarily submitting themselves to the psychiatric discipline of the state hospital care and consolation (Braslow and Starks 2005; Starks and Braslow 2005).

Unusual prior to World War II, voluntary admissions increased dramatically following the War (Grob 1973). In 1946, ten percent of patients voluntarily admitted themselves into California state hospitals (California 1946). Fourteen years later, voluntary admissions increased to 40 percent (California 1960: 2). Martha McFadden was typical of those individuals who freely chose hospitalization. Exhausted by caring for her four children and helping her husband run their farm, Martha fell into a deep depression and, in the fall of 1955, decided she desperately needed help. The twenty-three year old Martha told her admitting psychiatrist, “I’m here because I’m morbid and need treatment.”

The physician penned a sympathetic account of this young woman who, he wrote, felt deeply burdened by responsibilities to her children, husband, and their struggling farm. Married at 16 when she was pregnant with her first child, Martha had three more children in quick succession. By the time of her admission, Martha felt desperate hearing God’s voice saying that her husband was “going out with other women.” An even greater fear, according to the chart, “Patient is really afraid of being pregnant, the last pregnancy having occurred after husband had supposedly been sterilized. Patient was greatly upset by this, and now states that she would kill him and herself too before she has another baby.” She hoped that the hospital would provide her, at least, temporary shelter from her unrelenting exhaustion. Diagnosing her with a schizophrenic reaction, her physician wrote:

She has felt herself quite burdened, has been limited in her recreational and social activities by living on a farm…and has been feeling quite tired, even falling asleep during the day. She seems to want to deny to herself the privilege of some rest from this heavy work at home and seems very anxious in an obsessive sort of way to return to it. Obviously many of her defenses are obsessive in character. She has accepted the hospitalization as a situation where she can perhaps look at her problems with some distance and is just as extremely anxious about the complicated social setting in which she finds herself and so we are trying to very carefully orient her.

Martha ultimately rejected her physicians’ efforts to care for her. Though reluctantly, her mother and husband agreed to take her home. Elizabeth Tock, the ward physician, consented, though was not optimistic, writing, “This patient appears to be quite unstable at the present time, and is apparently a borderline schizophrenic with an acute schizophrenic episode prior to and at the time of admission.” Nevertheless, Dr. Tock had little choice. “Patient has requested her release from the hospital, and it is doubtful if further hospitalization would be helpful at this time, as the patient was obviously unreceptive. Patient may, therefore, leave when she wishes to do so.” Even though Martha remained ill, Dr. Tock accepted the limits of her power and her responsibilities. Institutional life did not imbue Dr. Tock with omnipotent powers to keep an ailing Martha against her will nor did it force Martha into submission.

I have used Martha’s case not to suggest that psychiatrists always acquiesced to their patients’ desires, though they often did. I am also not suggesting that state hospitals were necessarily (at least from the patients’ perspectives) safe and gentle refuges. But, even when patients experienced the more carceral, disciplinary nature of state hospitals, we can see a therapeutic rationale in what appears in hindsight as punitive and dehumanizing. Largely dictated by institutional imperatives to care for those unable to care for themselves, we can see a countervailing tendency.

Esther Erving’s comparatively long hospitalization illustrates the ways in which two seemingly incompatible forms of care—the “right hand” of disciplinary care and the “left hand” of therapeutic care, to analogize Pierre Bourdieu’s concepts of the Left and Right hands of the state (Loyal 2017)—constituted state hospital practices. Unlike Martha, Dr. Tock refused to discharge Esther despite her and her husband’s wishes. Also unlike Martha, Esther did not voluntarily become a patient but, instead, was forced kicking and screaming to become a Stockton State Hospital patient. Admitted in 1952, Esther first became psychotic while in a jail cell on a charge of theft. Barely arrested for a day, she began hearing the “voices of the Lord and holy spirits,” convinced a “spell” had been maliciously placed on her. The police quickly realized she belonged in a psychiatric hospital, had her examined by a court physician, and declared insane by a superior court judge, and then took her by police car to Stockton. The rocky start to her hospitalization was a portent of what lay ahead of her. Diagnosed with dementia praecox, she quickly descended into a psychotic hell as God’s voice turned menacing and the ward television began broadcasting intimate secrets about her life. A progress note just a month into her hospitalization read, “Pt. Is very hyperactive, singing and praying in a loud voice. Says she is an angel, God has taken possession of her body. Combative and resistive.” Electroconvulsive treatments fell far short in controlling her unpredictable attacks on unsuspecting patients and staff. “Special incident reports” chart her growing violence. One typical note describes Esther’s sudden, unprovoked ambush of a fellow patient: “Esther attacked her, bit her on the right hand, kicked her twice in the abdomen, started on her chest and hit her in the right eye. No apparent injury to Esther.” An hour later, Esther’s victim plaintively said “Esther is always so nice to me during the day, I can’t understand why she changes so quick.”

Over a hundred electroshock treatments, untold hours in restraints, and hundreds of hydrotherapy treatments did nothing to alter Esther’s increasingly unpredictably violent outbursts. Her husband’s desperation to have Esther returned to sanity, no doubt, added to the physicians’ sense of impotence. He wrote to her ward physician, Dr. Tock:

Esther is all I have in life…I love her to no end and there is nothing more that I would care for than to see her well physically and mentally….I know that it was beyond my power to know what to do in such a case as hers but I have always had faith in technical doctors because I feel that they evidently possess a greater ability to do more for people or else they would remain just a common doctor.

His faith and Dr. Tock’s and her colleagues’ desire to “do something,” even if a last resort, led to the decision to perform a transorbital lobotomy.

The lobotomy proved less than successful. A note shortly afterwards read, “Patient was started on EST since lobotomy because she was so combative with patients and unpredictable. Has to be watched all the time.” A note from July 1954 read, “Receives hydro, has maintenance with fair results. Seems to benefit more from seclusion.” Nine months later, after over 100 electroshock treatments, Esther’s physicians started her on Thorazine, hoping that they would not have to perform a more extensive lobotomy. “Took medicine reluctantly at first but is now taking it readily and seems more relaxed.” Six weeks later and prescribing 700 mg a day, physicians noted that “there has been some improvement.” The addition of Thorazine effectively quelled Esther’s doctors’ consideration of a more extensive second lobotomy. In fact, physicians at all California state hospitals abruptly ceased performing lobotomy once Thorazine become available. Nevertheless, despite the unusually large doses, the antipsychotic drug proved only partially successful and Esther again spiraled back into her unpredictable violent attacks on fellow patients and unsuspecting staff who passed within reach of her fists or feet. In what would become a similar pattern over the next few years, a new antipsychotic drug would be tried, often at hefty doses, with initial success and then, a few months later, Esther would lapse back into another round of unpredictable attacks on any one she believed had crossed her. This, in turn, would necessitate a try at a new antipsychotic drug, which invariably followed the same cycle of initial success followed by failure.

Throughout Esther’s hospitalization, her family remained involved in her care. They visited her often and took her home for extended leaves of absence. However, for Esther’s physicians, the successful elimination of her psychotic symptoms did not necessarily make her ready to permanently leave the hospital. Despite Esther’s husband’s and mother’s pleas, Dr. Tock steadfastly refused to discharge Esther, certain that Esther would tumble back into psychosis. Dr. Tock reminded the family that treatment involved much more than simply the taking of a drug. Esther’s care and treatment required the entire web of relationships and interventions that the hospital provided.

With the total embrace of its denizens, the state hospital provided the scaffolding for an expansive definition of disease and its treatment, where the social, psychological, and biological were legitimate explanations for as well as targets of therapeutics. The actual care, consolation, and effective treatment state hospital physicians provided for patients such as Martha, Esther, Claude and Albert stands in contrast to the narratives that depict state hospitals as horrors of an unenlightened past. At the same time, the logic of state hospital care that enabled these institutions to shelter those with serious mental illness also gave psychiatrists the instruments of control and domination that made interventions, such as lobotomy, legitimate treatments. Whether in the service of care and consolation or control and discipline, the same clinical vision shaped the care and treatment of Martha as well as Esther despite the dramatically different ways in which Dr. Tock treated the two patients. Dr. Tock’s clinical world view saw both patients and their diseases as embedded in a social context that was critical to each one’s care and treatment. In both cases, the asylum’s function required taking into account a patient’s wishes and desires and weighing that against the severity of the patient’s illness. When we consider Claude, Albert, Martha, and Esther together, we see a clinical vision that saw the social context and subjective meaning of psychosis as inextricably bound to understanding, treating, and caring for their patients. Control, discipline, alienation, empathy, consolation, care and treatment traveled together in State hospitals of the 1950s and 1960s. Perhaps the “right and left” sides of care were inherent in this system of care that defined disease based as much on violations of social norms as psychological distress. The state hospital as both a place of last resort and a place of refuge depended upon a larger political economy and set of ideological commitments, none of which survived intact beyond the 1960s.

## Part II: Retreat from the Social and Subjective (1970–1980)

### Deinstitutionalization

The psychiatric world changed quickly and dramatically from the late 1960s onward. Psychoanalysis quickly lost its hold over American psychiatry, replaced by a growing reliance on psychotropic drugs and a faith in biological reductionism. Most obvious, especially for those with serious mental illness, was the dismantling of the state hospital system. Over the course of less than three decades, the state hospital system and its logic of care and treatment became a bare whisper of its former self. The same political, economic, and ideological forces that attacked the welfare state also undid state governments’ commitment to provide comprehensive care for those unable to care for themselves. Armed with an ideology (largely unmoored from empirical evidence) of the virtues of community care, policymakers, politicians, activists, former patients, and clinicians all helped to dismantle the state hospital system. Ever since state governments began constructing asylums in the nineteenth century, the number of patients had steadily increased. The national state hospital resident population reached its zenith at 557,969 patients in 1955 (Natl. Inst. Ment. Health 1955). Following 1955, the state hospital population began a gradual decline. Following the 1965 passage of Medicaid and Medicare, state hospitals began a rapid free fall from a population of 475,000 in 1965 (Grob 1995) to 193,436 in 1975 (Clarke 1979; Goldman et al. 1983) and 92,000 in 1990. The state hospital population continued to fall in the new millennium: 50,000 in 2005 and then stabilizing at around 40,000 (Manderscheid et al. 2009).

California led the nation in emptying its state hospitals. Its hospital population peaked in 1955 (as did the nation as a whole), but fell more quickly than other states. In 1955, the resident state hospital population was 37,211 and, ten years later, had fallen to 25,674 (Calif. Legislative Analyst’s Office 2000, Natl. Inst. Ment. Health 1955). Ronald Reagan was sworn in as California’s governor on January 2, 1967. Elected on a wave of anti “big government” sentiment, a promise to slash taxes and end the spendthrift ways of his predecessor, Pat Brown (Putnam 2006), Reagan attacked the state hospital system as the quintessential Frankenstein of state largess run amok. During his eight-year tenure, the state hospital population underwent a freefall from 23,117 in 1967 to 6,299 in 1975 (Calif. Dep. Ment. Hyg. 1967, Calif. Legislative Analyst’s Office 2000).

The sustained and rapid decline after 1965 is mostly accounted for by the passage of Medicaid and Medicare, which allowed state hospitals to discharge a large number of elderly patients to nursing homes. In 1972 Congress amended the Social Security Act, expanding the eligibility criteria of those covered by the Supplemental Security Income to the Aged, the Disabled, and the Blind (known more commonly as SSI). This entitlement program provided stable income for those with serious and persistent mental illness who were ineligible for Medicare and Social Security Disability Income (SSDI). This expansion maintained the sustained fall in resident population, allowing state hospital physicians to discharge patients into the community—though, as became increasingly apparent, into lives that were often far more brutal, isolating, and impoverished than they had experienced in state hospitals (Frank and Glied 2006).

While no aspect of state hospital life remained untouched by this depopulation, lengths of stay changed dramatically as physicians were placed under increasing pressure to discharge patients as quickly as possible. No longer could psychiatrists decide to keep a patient hospitalized solely based on what they deemed clinically necessary—though, as we will soon see, the nature of what physicians believed was “clinically necessary” changed, turning administrative edicts into transformed and significantly narrowed clinical vision. In 1960, for example, for the 13,154 patients discharged in fiscal year 1960, the median hospital stay was 2.5 months (California 1960). By 1975, this had plummeted to 17 days (Chandler and Sallychild 1977). As patients stayed for shorter and shorter periods of time psychiatrists focused on a narrowing set of signs and symptoms.

### A new Clinical Vision

Jonathan Harrison’s numerous hospitalizations from the late 1960s through the 1970s give us a glimpse into a new clinical vision made necessary by the rapidly changing conditions of clinical care. Let us start with one of his earliest hospitalizations. It lasted barely two weeks, beginning the week before Christmas of 1969. At the time of his admission, Jonathan lived in a makeshift shelter of discarded lumber and dismembered cardboard boxes that he had “constructed” after his last discharge a few months earlier. The admission note reads as follows:

Subject was observed barking at passing motorists in the manner of a dog. He was on all fours. He had been brought into the General Hospital but walked away prior to treatment. He seemed to be attempting to pass himself off as a dog. Rubbing against a pole. Subject seems to be suffering from hallucinations of impending tragedy. According to the police, patient is a danger to himself and others.

Address of patient: Transient.

Jonathan’s case file chronicles the waxing and waning of his profoundly psychotic symptoms and his growing alienation from his family and those around him. But, unlike the patient histories we encountered previously, Jonathan’s psychiatrist does not provide a causal narrative of events that led his psychosis. Instead, the psychiatrist writes as if Jonathan’s disease had autonomously unfolded, unaffected by Jonathan’s life history or psychology. We do learn that he was fully aware that he was not a dog. Instead, he was hoping to fool those who were trying to harm him into mistaking him for dog. Yet, we learn nothing of the events leading up to his paranoid delusions, why he believed he was being pursued, or why he had chosen such an odd strategy of evasion. For the psychiatrist, what mattered was the fact he was delusional. The content of the delusion was irrelevant just as were the details of his auditory hallucinations. Jonathan’s signs and symptoms only mattered as signifiers of underlying disease rather than mysteries with meaning waiting to be probed and unraveled though psychotherapy.

The psychiatrist does give a brief, perfunctory catalogue of Jonathan’s social history. He writes of Jonathan’s early childhood, “no significant traumas.” He gives equally short shrift to the remainder of his psychosocial development. The psychiatrist does note that Jonathan has no residence, which will become an ever-present hell for Jonathan as he careens over the next few years between the streets, jail, and hospitals. It was a peripatetic whirlwind fueled by his psychosis that temporarily and episodically went underground during his hospitalizations as his physicians plied him with antipsychotic drugs.

### Antipsychotic Drugs in Daily Practice, Part 2: Affirming the Biological

Jonathan’s discharge summary illustrates the extent to which the nature of disease, treatment, and the goals of hospitalization looked very different than they did a decade earlier.

Course in the Hospital: The patient was placed on substantial amounts of chlorpromazine (1,600 mg) to control his agitation and make him bearable by the other patients. He would be up all night rummaging through their lockers and generally behaving in a rather bizarre manner. Thorazine rapidly reduced this. The patient, although still obvious mentally ill, was able to understand that he needed treatment and he was allowed to change to voluntary status at the end of the 72 hours. On several subsequent occasions, he came in and indicated he wanted to sign out, and he was informed that we did not feel he was ready…The acute aspects of his illness subsided steadily, however, the chronic degree of illness remained substantially high, and it is obvious that this patient will remain a bizarre, chronically schizophrenic individual with intermittent aggravations leading him to repeated hospitalizations. Attempts to contact the family indicated that he was not welcome with the family in any way.

Diagnosis: schizophrenia, chronic undifferentiated type.

Recommendations: (1) Condition improved. The patient remains chronically schizophrenic with maximum hospital benefit. (2) Refer to mental health clinic. (3) A 14-day supply of medicine was sent with the patient.

There are several points worth emphasizing. First, the psychiatrist’s focus on his psychotic symptoms and their response to chlorpromazine illustrates a substantially narrowed perspective where signs and symptoms speak for themselves and do not have much more meaning than pointing towards his schizophrenia. Second, the large chlorpromazine dose achieved over the course of just a handful of days suggests that the psychiatrist increased the dose as rapidly as possible to hasten Jonathan’s discharge. In other words, the signs and symptoms that respond to chlorpromazine were the main focus of treatment and their successful elimination was the criterion by which Jonathan was discharged.

Third, and related to the rapid escalation of the dose, we see that the doctor has articulated a radically diminished horizon for psychiatric treatment compared to patients admitted a few years earlier: “The acute aspects of his illness subsided steadily, however, the chronic degree of illness remained substantially high, and it is obvious that this patient will remain a bizarre, chronically schizophrenic individual with intermittent aggravations leading him to repeated hospitalizations.” The very definition of successful treatment has dramatically changed. Jonathan’s psychiatrist measured successful treatment based on eliminating a narrow set of drug-responsive signs and symptoms. Despite remaining psychotic (though no longer agitated) and facing certain homelessness, Jonathan was declared to have achieved “maximum hospital benefit.” This assessment rested upon a fundamentally new clinical vision where the social world has receded into the background in the clinical calculus of Jonathan’s psychiatrist.

**Fig. 2:**
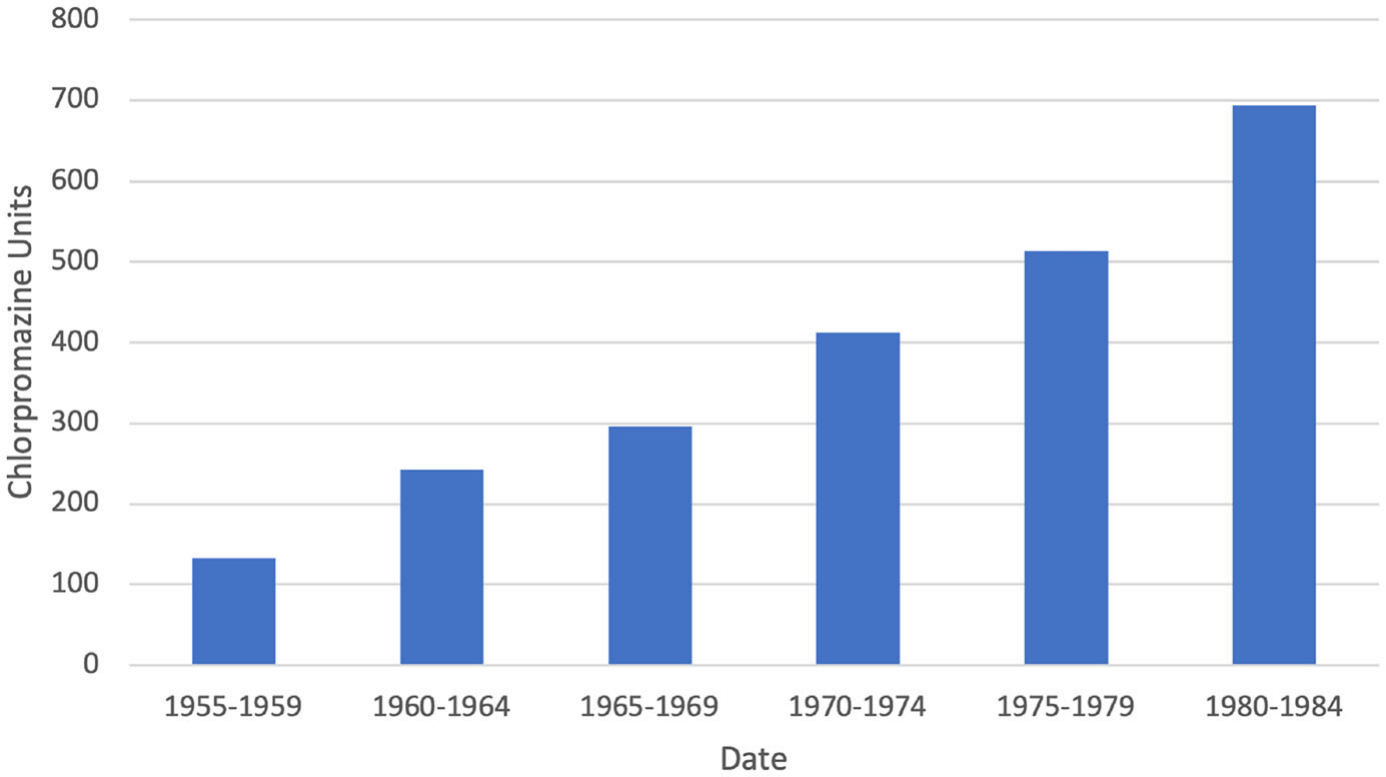
Chlorpromazine Equivalence Units, 1955–1985

The escalation of antipsychotic drug dosages as hospital lengths of stay shortened was a widespread phenomenon. Based on the random sample of patients described in Sect. 1, Fig. 2 shows the escalation of dosage for patients diagnosed with psychotic disorders between 1955 and 1985. To normalize the dosages of the various antipsychotic drugs used over those three decades, I have converted the doses into chlorpromazine equivalence units (Andreasen et al. 2010) (Andreasen et al. 2010). As psychiatric beds became increasingly scarce, and lengths of stay shortened, psychiatrists continued to escalate antipsychotic doses. This created a self-fulfilling and self-reinforcing cycle in which the limited signs and symptoms that antipsychotic drugs treated defined psychosis and its medical treatment. Simultaneously, the social and experiential slipped further and further into the background. The spectacular success of the neurosciences aided and abetted this shrinking clinical vision, adding to a belief that psychiatry was finally making good on its 100-year promissory note to make psychiatry a brain science.

As Jonathan’s psychiatrist predicted, Jonathan continued to cycle in and out of the hospital throughout the 1970s. And, as hospital beds became increasingly scarce, his hospitalizations shortened, further narrowing what psychiatrists meant by the phrase “maximal hospital benefit.” In addition to a prescription, Jonathan would be given an appointment to the public mental health clinic as he exited the hospital. Neither proved useful to him since he rarely, if ever, took the prescribed medication and rarely, if ever, kept his outpatient mental health appointments. Not surprisingly, having been unable to find work and having completely exhausted his family, Jonathan returned to live in whatever makeshift shelter he could either find or piece together with the flotsam and jetsam he collected near where he called home in Downtown Los Angeles. Once he began collecting SSI checks, he interspersed stretches of homelessness with a week or so in a single room occupancy hotel.

Over the course of the decade, Jonathan’s “institutional circuit” (Hopper 2003) expanded to include frequent incarcerations for a variety of misdemeanors, such as urinating in public, disturbing the peace, and trespassing. As the decade wore on and hospital beds evaporated, it became increasingly unlikely when police brought a psychotic, disruptive individual to an emergency room that physicians would admit him or her. This often left police with the difficult choice of either taking doing nothing, taking the psychotic individual to an emergency room with little likelihood of her or him being hospitalized, or arresting and then jailing the individual, certain they had, at least temporarily, solved the problem of a psychotic individual potentially harming himself or herself or others. When Jonathan’s psychosis would reach such a crescendo that he came to the attention of the police, they often chose to arrest him, a much easier and a more reliable solution than dragging him to the nearest emergency room where he would most likely be discharged back to the streets rather then admitted to a psychiatric ward. Throughout the 1970s, Jonathan tumbled from hospital, to homelessness, to SRO, to jail and back again to the hospital, only to begin the tumultuous circuit all over again once his psychiatrists deemed he had reached “maximum hospital benefit.” Jonathan’s ultimate fate is unknown. His last admission lasted 3 days and on discharge he was given a 10-day supply of a new antipsychotic drug, Mellaril (thioridazine). The last entry reads, “TREATMENT COMPLETED (IMPROVED).”

### Social and Psychological Amnesia

Psychiatrists in the 1950s and early 1960s had a radically different understanding of “maximum hospital benefit” than psychiatrists in the 1970s and 1980s. These differences fundamentally turned on which signs and symptoms psychiatrists saw as important, and then the ways in which they made sense of these signs and symptoms in relationship to the patient’s disease. I have tried to suggest that the “total institution” of the 1950s was not nearly as harsh, totalizing, and dehumanizing as we might have believed. Under the right historical circumstances—such as post-War America with its growing faith in the Keynesian state—state hospitals managed to create a therapeutic space, however imperfect. For better or worse, state hospital psychiatrists saw a world refracted through a lens in which they had comprehensive responsibility for their charges. Even a partial list of their most important interventions illustrates the complexity of their worldview: lobotomy, electroshock, psychotherapy, industrial therapy, occupational therapy, recreational therapy, bibliotherapy, music therapy, and beauty shop therapy. For state hospital psychiatrists, if subjective experiences mattered, it was because the social structure of the hospital made this possible.

With the rapid emptying of state hospitals in the late 1960s and early 1970s, psychiatrists had little choice but to redefine the meaning of “maximum hospital benefit” and focus increasingly on a narrow set of symptoms that antipsychotic drugs could alleviate. This new world was not of their own making, nor was it the making of antipsychotic drugs. Deinstitutionalization and the delegitimization of comprehensive care for a disease that, by definition, is a failure to function, had little to do with scientific evidence or the effectiveness of psychotropic drugs. Rather, it had everything to do with the destruction of the welfare state, abandonment of Keynesian economic policy, and the shift towards neoliberal economics and policy. Left in the wake of these forces are a fragmented social safety net, impoverished families unable to care for their psychotic loved ones, and a rhetorical embrace of a barely existing and unrealized community care system. These are now the building blocks of the practical, everyday way of understanding and treating individuals with serious mental illness.

Just as psychotropic drugs did not transform treatment in the 1950s and 1960s, we should not be too quick to blame psychiatrists’ reliance on them as the engine that has led to an increasingly biological reductionist clinical psychiatry. Admittedly, clinical psychiatry’s embrace of psychopharmacology and biological reductionism has functioned as clinical blinders, making patient’s lived experience increasingly irrelevant to clinical psychiatric practice. But contingent historical circumstances, as this history suggests, made the turn towards biological reductionism more of a marriage of convenience than a choice based on scientific evidence. Since the 1970s, psychiatrists increasingly have found themselves with little more to offer their patients than a prescription.

It almost goes without saying that psychiatric disease is simultaneously social, biological, psychological, and experiential. Yet, this history illustrates that psychiatrists’ clinical beliefs and treatments were (and are) as dependent on the contingencies of social life as are their patients’ psychiatric disorders. Psychiatry may not own the current crisis of inequality that has plunged tens of thousands into despondency, but it owns a part in addressing it. First, though, the profession must reckon with its myths, assumptions, and shibboleths—learning from its history, rather than repeating it.

## Footnotes

1 All patient cases cited are from Stockton State Hospital and Camarillo State Hospital. The use of these patient records was approved by the State of California Human Subjects Institutional Review Board. All patient names and identifying data have been changed to protect patient confidentiality. Should readers have interest in any specific cases cited, they may write to the California Department of Mental Health for permission to review the cases.
